# Associations Between Sleep-Related Characteristics and Mind Wandering in Adults with ADHD: A Cross-Sectional Study with a Control Group

**DOI:** 10.3390/bs16081372

**Published:** 2026-08-10

**Authors:** Samet Kaya, Fatih Ekici, Yavuz Selvi, Ali Kandeğer

**Affiliations:** 1 Department of Psychiatry, Bunyan State Hospital, Kayseri 38600, Türkiye; samet.kaya4@saglik.gov.tr; 2 Department of Psychiatry, Faculty of Medicine, Selçuk University, Konya 42250, Türkiye; fatih.ekici@selcuk.edu.tr (F.E.); dryavuzselvi@selcuk.edu.tr (Y.S.)

**Keywords:** attention-deficit/hyperactivity disorder, adult ADHD, excessive mind wandering, mid-sleep time, subjective sleep quality, sleep–wake cycle

## Abstract

Excessive mind wandering is increasingly recognized as a clinically relevant cognitive feature of adult attention-deficit/hyperactivity disorder (ADHD), yet its relationship with sleep-related characteristics remains insufficiently understood. This cross-sectional study with a control group examined associations between sleep-related characteristics and excessive mind wandering in 316 adults with clinically confirmed ADHD and 121 screened controls. Participants completed measures of ADHD symptoms, mind wandering, anxiety–depressive symptoms, and self-reported sleep-related characteristics adapted from Pittsburgh Sleep Quality Index items; mid-sleep time was calculated from reported sleep onset and final awakening. Group comparisons, correlation analyses, and multiple regression analyses were conducted. Adults with ADHD showed higher mind wandering, greater ADHD symptom severity, anxiety–depressive symptom burden, poorer subjective sleep quality, and later mid-sleep time than controls, whereas sleep duration did not differ between groups. Mind wandering was associated with ADHD symptom severity, anxiety–depressive symptoms, later mid-sleep time, and poorer subjective sleep quality. In regression analysis, ADHD diagnosis was the strongest independent correlate of mind wandering. Anxiety–depressive symptom burden, poorer subjective sleep quality, and later mid-sleep time, but not sleep duration, also remained independently associated with higher mind wandering. Findings suggest that sleep timing and subjective sleep quality may be clinically relevant to excessive mind wandering in adults with ADHD.

## 1. Introduction

Attention-deficit/hyperactivity disorder (ADHD) is a common neurodevelopmental disorder characterized by persistent symptoms of inattention, hyperactivity, and impulsivity (Kooij et al., 2019). Although ADHD begins in childhood, symptoms frequently persist into adulthood, often with changes in clinical presentation rather than complete remission (Adler et al., 2017; Fayyad et al., 2017). In adults, overt motor hyperactivity may become less prominent and may instead manifest as inner restlessness, cognitive overactivity, and difficulties in self-regulation. In addition to the core diagnostic symptoms, adult ADHD is commonly accompanied by clinically relevant features such as emotional dysregulation and sleep–wake disturbances, both of which may substantially contribute to functional impairment (Bozhilova et al., 2018; Soler-Gutiérrez et al., 2023; Surman & Walsh, 2021).

Increasing evidence suggests that ADHD is not only associated with increased susceptibility to external distraction, but also with difficulty regulating internally generated thought (Bozhilova et al., 2018; Helfer et al., 2019; Osborne et al., 2023). Within this framework, excessive mind wandering (EMW) has emerged as a potentially important cognitive feature of adult ADHD (Mowlem et al., 2019a). Mind wandering refers to a shift of attention away from an ongoing task toward internal thoughts that are unrelated to the task at hand (Kandeğer et al., 2024; Seli et al., 2015). Although mind wandering is common in the general population, it appears to be more frequent, less controllable, and more functionally impairing in individuals with ADHD, thereby taking the form of EMW (Dekkers et al., 2025; Mowlem et al., 2019b). Prior studies have shown that EMW is positively associated with ADHD symptom severity and functional impairment, suggesting that it represents a meaningful dimension of the adult ADHD phenotype (Biederman et al., 2019; Bozhilova et al., 2018).

The clinical expression of excessive mind wandering may also vary according to ADHD symptom profile and developmental stage (Babinski, 2024). As overt hyperactive–impulsive symptoms often become less prominent in adulthood, excessive mind wandering may be particularly relevant to persistent difficulties in sustaining attention, regulating internally generated thought, and maintaining engagement during low-stimulation activities, while also reflecting impaired inhibitory control (Bozhilova et al., 2018). Sex may represent an additional source of heterogeneity, although previous findings regarding sex differences in excessive mind wandering among adults with ADHD have been limited and inconsistent (Babinski, 2024; Mowlem et al., 2019a). Examining excessive mind wandering across ADHD presentations and sexes may therefore clarify whether it represents a general feature of adult ADHD or is more closely related to particular symptom dimensions.

A plausible neurocognitive account of EMW in ADHD has been proposed in studies of large-scale brain networks. Neuroimaging studies have shown reduced activation in executive control regions, particularly the inferior frontal cortex and anterior cingulate cortex, together with insufficient suppression of the default mode network (DMN) during task performance (Duffy et al., 2021; Hart et al., 2013; Rubia, 2018). Persistent activity within DMN regions, including the posterior cingulate cortex and medial prefrontal cortex, may interfere with sustained externally directed attention and facilitate internally oriented thought. Accordingly, EMW has been conceptualized as a behavioral correlate of impaired coordination between executive control networks and the DMN in ADHD (Bozhilova et al., 2018; Cortese et al., 2012; Hart et al., 2014).

Sleep-related complaints are also highly prevalent in adults with ADHD. Previous studies have reported increased rates of eveningness, delayed sleep timing, insomnia symptoms, and other sleep-related problems in this population compared with the general population (Luu & Fabiano, 2025; van der Ham et al., 2024; Wynchank et al., 2017). Adults with ADHD tend to report later bedtimes and wake times, and actigraphy- and melatonin-based studies suggest that these patterns may reflect delayed circadian timing in at least a subgroup of patients (Snitselaar et al., 2017; Van Veen et al., 2010). From a clinical perspective, mid-sleep time may serve as a pragmatic indicator of sleep timing and may capture a dimension of sleep–wake regulation that is not reflected by sleep duration alone.

Sleep–wake regulation is closely linked to executive functioning, arousal, and the balance between externally directed attention and internally generated cognition (Schmidt et al., 2007; Surman & Walsh, 2021). Delayed sleep timing and circadian misalignment may weaken cognitive control and increase vulnerability to spontaneous, task-unrelated thought, particularly under conditions of suboptimal arousal. A recent systematic review suggested that poorer sleep is associated with spontaneous cognition, including mind wandering, although the findings remain heterogeneous (Cárdenas-Egúsquiza & Berntsen, 2022). In adult ADHD, this question may be especially relevant because both EMW and sleep–wake disturbances are common, yet their joint relationship remains insufficiently characterized. In particular, it remains unclear whether sleep timing is associated with EMW independently of total sleep duration and subjective sleep quality.

Therefore, the present cross-sectional study with a control group examined the association between EMW and sleep–wake cycle indicators in adults with ADHD and controls. We specifically investigated whether mid-sleep time was associated with EMW beyond total sleep duration and subjective sleep quality. We hypothesized that adults with ADHD would show higher levels of EMW than controls, that EMW would be positively associated with ADHD symptom severity, and that later mid-sleep time would be associated with greater EMW. We further expected mid-sleep time to remain independently associated with EMW after adjustment for sleep duration and sleep quality.

## 2. Methods

### 2.1. Study Setting

This cross-sectional study with a control group was conducted at the Adult Neurodevelopmental Disorders Outpatient Clinic of Selçuk University, Konya, Türkiye. The clinic is a specialized tertiary referral unit for the assessment and follow-up of adults with attention-deficit/hyperactivity disorder (ADHD) and serves a large catchment area in Central Anatolia.

### 2.2. Participants and Procedure

The clinic follows a structured diagnostic pathway consistent with the Adult ADHD Assessment Quality Assurance Standard proposed by the United Kingdom Adult ADHD Network (Adamou et al., 2024). Adults evaluated in the general psychiatry outpatient service at Selçuk University Hospital in Konya and referred with suspected ADHD underwent a comprehensive diagnostic assessment at the Adult Neurodevelopmental Disorders Outpatient Clinic. The assessment was conducted by experienced psychiatrists and, when available, included collateral developmental information from family members or close informants. In accordance with the clinic’s standard procedure, the diagnostic assessment consisted of at least two sessions, each lasting a minimum of 60 min.

ADHD diagnosis was established according to DSM-5 criteria on the basis of developmental history, current symptom profile, functional impairment, age at onset, persistence of symptoms across settings, and differential diagnostic evaluation. Childhood ADHD symptoms were retrospectively assessed as part of the developmental history, with particular attention to symptom onset before the age of 12, persistence across developmental periods, and impairment across settings. The Structured Clinical Interview for DSM-5 Disorders–Clinician Version (SCID-5-CV) was used to assess current and lifetime psychiatric disorders, psychiatric comorbidities, and exclusionary diagnoses. A structured sociodemographic and clinical data form was also completed during the clinical evaluation.

After the diagnostic assessment, eligible participants received an online link to the self-report questionnaire battery prepared for the study. Only individuals with a clinically confirmed diagnosis of ADHD who completed all study measures were included in the ADHD group.

Participants were excluded if they had intellectual disability, autism spectrum disorder, neurocognitive disorder, psychotic disorder, non-remitted bipolar disorder, or current substance use disorder. Psychotic disorders, bipolar disorder, and substance use disorders were systematically assessed using the SCID-5-CV. Conditions not directly covered by the SCID-5-CV, including intellectual disability, autism spectrum disorder, and neurocognitive disorders, were evaluated according to DSM-5 criteria through developmental history, assessment of cognitive and functional profiles, and clinical interview.

A total of 316 adults with clinically confirmed ADHD completed the study battery and were included in the ADHD group. Current ADHD medication use and psychiatric comorbidities were not exclusion criteria, as both are common in adult ADHD and their exclusion would have reduced the clinical representativeness of the sample. These variables were recorded during the clinical assessment.

Typically developing controls were recruited on a voluntary basis through participation announcements distributed via university mailing lists, departmental networks, and messaging groups. Recruitment was not consecutive, and no individual matching procedure was used. To reduce overrepresentation from any single academic discipline, efforts were made to include volunteers from a range of university departments and backgrounds. All volunteers underwent psychiatric screening using the SCID-5-CV. Individuals were excluded if they had any current or lifetime psychiatric disorder, including remitted disorders, hazardous alcohol use, current or past substance use disorder, or current psychotropic medication use. Participants with incomplete questionnaire data were excluded from the analyses. The final sample consisted of 437 participants: 316 adults with ADHD and 121 controls.

The study protocol was approved by the Local Ethics Committee of Selçuk University with decision number 2023/420. All participants provided written informed consent before participation. Participation was voluntary, and all data were collected and stored anonymously in accordance with the Declaration of Helsinki.

### 2.3. Measures

All participants completed a standardized self-report battery consisting of a sociodemographic and clinical data form, the Adult ADHD Self-Report Scale, the Mind Excessively Wandering Scale, and the Hospital Anxiety and Depression Scale and self-report sleep-related questions.

#### 2.3.1. Sociodemographic and Clinical Data Form

The sociodemographic and clinical data form was used to collect information on age, sex, years of education, psychiatric history, psychiatric comorbidities, current and previous medication use, alcohol and substance use history, and sleep–wake characteristics. Clinical information was obtained during the diagnostic interview and supplemented by self-report data when appropriate.

#### 2.3.2. Assessment of ADHD Symptom Severity

ADHD symptom severity was assessed using the Adult ADHD Self-Report Scale (ASRS-v1.1), developed by Kessler et al. (2005). The ASRS is an 18-item self-report instrument based on DSM-IV-TR ADHD criteria and comprises two subscales: inattention (items 1–9) and hyperactivity/impulsivity (items 10–18). Higher scores indicate greater ADHD symptom severity (Kessler et al., 2005).

The Turkish version of the ASRS has demonstrated adequate validity and reliability in previous studies. Its internal consistency has been reported as high, with a Cronbach’s alpha of 0.88. A cut-off score of 30 has been recommended, with a sensitivity of 0.75, specificity of 0.79, kappa value of 0.44, positive predictive value of 0.46, and negative predictive value of 0.93 (Evren et al., 2016). In the present study, the ASRS total score was used in the analyses.

#### 2.3.3. Assessment of Excessive Mind Wandering

Excessive mind wandering was assessed using the Mind Excessively Wandering Scale (MEWS), developed by Mowlem et al. (2019b). The MEWS is a 12-item Likert-type self-report scale designed to evaluate excessive and difficult-to-control internal mentation, particularly in adults with ADHD (Mowlem et al., 2019b).

Higher scores indicate greater difficulty in mental control and higher levels of excessive mind wandering. The Turkish version of the MEWS has been shown to be valid and reliable in adult ADHD samples. In the Turkish validation study, the scale demonstrated good internal consistency, with a Cronbach’s alpha of 0.874 (Aksoy et al., 2022). In the present study, the MEWS total score was used in the analyses.

#### 2.3.4. Assessment of Sleep Parameters

Sleep-related data were obtained based on participants’ self-reports. Participants provided information regarding their typical sleep onset time, wake-up time, and average sleep duration. The reported sleep data were intended to reflect participants’ habitual sleep patterns. Total sleep duration was calculated by subtracting sleep onset time from wake-up time. Mid-sleep time was defined as the midpoint between sleep onset and final awakening and was calculated by adding half of the total sleep duration to the reported sleep onset time.

Subjective sleep quality was assessed using a single-item Likert-type measure ranging from 0 (very poor) to 10 (very good), allowing for a broader evaluation of participants’ overall sleep experience.

The sleep parameters assessed in this study conceptually overlap with components of the Pittsburgh Sleep Quality Index (PSQI); however, the standard PSQI global score and component scores were not calculated (Buysse et al., 1989). Instead, sleep-related questions were constructed based on the PSQI framework to enable a practical and focused assessment of the variables of interest. These study-specific questions were used to assess distinct sleep-related characteristics rather than to form a single abbreviated version of the PSQI. Because they were not intended to constitute a unified scale, no internal consistency analysis was undertaken. Sleep duration variables were converted into decimal hours for statistical analyses.

#### 2.3.5. Assessment of Anxiety and Depressive Symptoms

Anxiety and depressive symptoms were assessed using the Hospital Anxiety and Depression Scale (HADS), developed by Zigmond and Snaith (1983). The HADS is a 14-item Likert-type self-report scale comprising two subscales: anxiety and depression (Zigmond & Snaith, 1983). The HADS was selected because it provides a brief assessment of anxiety and depressive symptom burden while placing relatively limited emphasis on somatic symptoms, thereby reducing potential overlap with sleep-related complaints and other physical symptoms.

The Turkish version of the HADS has demonstrated acceptable validity and reliability, with an internal consistency (Cronbach’s alpha) of 0.818 (Aydemir et al., 1997). In the present study, the HADS total score was used as a composite index of anxiety–depressive symptom burden and was included as a covariate in the regression analyses.

### 2.4. Statistical Analysis

Statistical analyses were performed using IBM SPSS Statistics version 22.0. Continuous variables were summarized as means and standard deviations, and categorical variables were presented as frequencies and percentages. The distribution of continuous variables was evaluated using the Kolmogorov–Smirnov test, together with visual inspection of histograms and Q–Q plots. Between-group comparisons were conducted using independent-samples *t*-tests for continuous variables and chi-square tests for categorical variables. Effect sizes for group differences in continuous variables were reported using Cohen’s d. Pearson correlation analyses were performed separately in the ADHD and control groups to examine associations among ADHD symptom severity, excessive mind wandering, anxiety–depressive symptom burden, and sleep–wake variables.

To identify independent correlates of excessive mind wandering, a multiple linear regression analysis was conducted in the total sample, with the MEWS total score entered as the dependent variable. Diagnostic group, sex, years of education, HADS total score, subjective sleep quality, total sleep duration, and mid-sleep time were entered as predictors. Diagnostic group was coded as 0 for controls and 1 for ADHD. Years of education was included because it differed significantly between groups. Multicollinearity was evaluated using tolerance and variance inflation factor values. Diagnostic group was prioritised in the primary model because the analysis was designed to evaluate the independent association of clinically established ADHD status with excessive mind wandering, whereas ADHD symptom severity was examined separately as a dimensional correlate. Statistical significance was set at two-tailed *p* < 0.05.

As an exploratory secondary analysis, adults with ADHD who were currently using ADHD medication were compared with those not using ADHD medication in terms of ADHD symptom severity, excessive mind wandering, subjective sleep quality, total sleep duration, and mid-sleep time. Medication status was assessed separately because it was applicable exclusively to the ADHD group and was not recorded in sufficient detail to support reliable adjustment in the primary pooled model.

## 3. Results

### 3.1. Sample Characteristics and Group Comparisons

A total of 437 participants were included in the study: 316 adults with ADHD and 121 controls. Women constituted 54.9% of the total sample. The ADHD and control groups did not differ significantly in age or sex distribution; however, the control group had significantly longer education than the ADHD group (*p* < 0.001). Group comparisons are presented in Table 1.

As expected, adults with ADHD had significantly higher ASRS and MEWS scores than controls, with large effect sizes across ADHD-related symptom measures and excessive mind wandering indices. The ADHD group also showed significantly higher HADS total scores than controls.

Regarding sleep–wake variables, the ADHD group reported lower subjective sleep quality and later mid-sleep time than the control group. In contrast, mean daily sleep duration did not differ significantly between groups. Within the ADHD group, psychiatric comorbidity was common, and 116 participants (36.7%) were currently receiving ADHD medication.

In exploratory analyses within the ADHD group, current ADHD medication use was not associated with significant differences in ASRS total score, MEWS score, subjective sleep quality, total sleep duration, or mid-sleep time (all *p* > 0.05). Medication status was assessed cross-sectionally as a binary variable; therefore, dose, treatment duration, adherence, and medication timing were not evaluated.

### 3.2. Correlation Analyses

Pearson correlation analyses were conducted separately in the ADHD and control groups (Table 2). In the ADHD group, MEWS scores were moderately and positively correlated with ASRS total scores (r = 0.57, *p* < 0.001). MEWS scores were also positively associated with HADS total scores (r = 0.26, *p* < 0.001) and mid-sleep time (r = 0.15, *p* = 0.008), and negatively associated with subjective sleep quality (r = −0.18, *p* = 0.001). No significant association was observed between MEWS scores and total sleep duration.

A similar pattern was observed in the control group. MEWS scores were positively correlated with ASRS total scores (r = 0.79, *p* < 0.001), HADS total scores (r = 0.47, *p* < 0.001), and mid-sleep time (r = 0.38, *p* < 0.001), and negatively correlated with subjective sleep quality (r = −0.41, *p* < 0.001). Total sleep duration was not significantly associated with MEWS scores in the control group.

Formal comparisons of the corresponding correlation coefficients using Fisher’s r-to-z transformation showed that the associations of MEWS scores with ASRS total scores (z = −3.92, *p* < 0.001), HADS total scores (z = −2.26, *p* = 0.024), subjective sleep quality (z = 2.35, *p* = 0.019), and mid-sleep time (z = −2.30, *p* = 0.021) differed significantly between the ADHD and control groups. Although the direction of these associations was consistent across groups, the correlations were stronger in the control group (Table 3).

### 3.3. Multiple Linear Regression Analysis

To examine the independent correlates of excessive mind wandering, a multiple linear regression analysis was conducted in the total sample using MEWS total score as the dependent variable (Table 4). Diagnostic group, sex, years of education, HADS total score, subjective sleep quality, total sleep duration, and mid-sleep time were entered as predictors. The model was statistically significant, F(7, 429) = 68.55, *p* < 0.001, and explained 52.8% of the variance in MEWS scores (R^2^ = 0.528, adjusted R^2^ = 0.520).

Diagnostic group emerged as the strongest independent correlate of MEWS scores (β = 0.544, *p* < 0.001), indicating that ADHD diagnosis was associated with higher levels of excessive mind wandering after adjustment for demographic, psychological, and sleep-related variables. Higher HADS total scores were also independently associated with higher MEWS scores (β = 0.207, *p* < 0.001).

Among the sleep–wake variables, poorer subjective sleep quality was independently associated with higher MEWS scores (β = −0.109, *p* = 0.007), whereas later mid-sleep time was independently associated with higher MEWS scores (β = 0.074, *p* = 0.048). Total sleep duration was not a significant predictor of MEWS scores (*p* = 0.381). Sex and years of education were not significant independent predictors.

Overall, these findings indicate that excessive mind wandering is associated not only with ADHD diagnosis and anxiety–depressive symptom burden, but also with sleep timing and subjective sleep quality. In contrast, sleep duration did not show an independent association with excessive mind wandering.

## 4. Discussion

This cross-sectional study examined the relationship between excessive mind wandering, ADHD symptom severity, and sleep–wake cycle indicators in adults with ADHD compared with a control group. The main findings were threefold. First, adults with ADHD showed substantially higher levels of excessive mind wandering than controls, and excessive mind wandering was positively associated with ADHD symptom severity. Second, adults with ADHD reported later mid-sleep time and poorer subjective sleep quality, whereas total sleep duration did not differ between groups. Third, mid-sleep time and subjective sleep quality were independently associated with excessive mind wandering after adjustment for diagnostic group, education, sex, anxiety–depressive symptom burden, and sleep duration. These findings suggest that excessive mind wandering in adult ADHD is related not only to ADHD diagnosis and psychological symptom burden, but also to the timing and perceived quality of sleep.

The higher level of excessive mind wandering observed in the ADHD group is consistent with the view that ADHD involves difficulties not only in resisting external distraction, but also in regulating internally generated thought. Excessive mind wandering may be related to the persistent inattentive and internally experienced features of adult ADHD, while difficulties in inhibitory control may also contribute to its clinical expression (Bozhilova et al., 2018). Potential variation across ADHD presentations and sexes should therefore be considered when interpreting excessive mind wandering as a feature of adult ADHD (Babinski, 2024; Mowlem et al., 2019a). Previous studies have shown that excessive mind wandering is common in adults with ADHD and is associated with symptom severity and functional impairment (Biederman et al., 2019; Bozhilova et al., 2018; Mowlem et al., 2019b).

Our findings support this literature by showing a robust association between MEWS scores and ASRS total scores. Although this and the associations with anxiety–depressive symptoms, subjective sleep quality, and mid-sleep time were stronger in the control group, their direction was consistent across groups. These relationships may therefore extend beyond clinically diagnosed ADHD, while restricted score ranges and greater clinical heterogeneity may partly explain the weaker correlations within the ADHD group (Katzman et al., 2017; Mowlem et al., 2019a). The marked elevation of MEWS scores in the ADHD group further suggests that excessive mind wandering may capture a clinically meaningful cognitive dimension of adult ADHD, particularly the experience of uncontrolled, intrusive, and difficult-to-disengage internal mentation (Biederman et al., 2019; Bozhilova et al., 2018).

The most distinctive finding of the present study concerns the sleep–wake cycle. Although total sleep duration was not independently associated with excessive mind wandering, later mid-sleep time and poorer subjective sleep quality were. This pattern suggests that the relationship between sleep and excessive mind wandering may be better explained by sleep timing and sleep experience than by sleep quantity alone. This is clinically relevant because adults with ADHD frequently report delayed sleep timing, eveningness, insomnia symptoms, and irregular sleep–wake patterns (Bijlenga et al., 2019; Coogan & McGowan, 2017; Surman & Walsh, 2021; Wynchank et al., 2017). In this context, mid-sleep time may provide a useful clinical marker of delayed sleep timing, although it should not be interpreted as a direct measure of circadian phase.

The absence of an independent association between total sleep duration and excessive mind wandering is also noteworthy. A recent systematic review suggested that sleep outcomes and spontaneous cognition, including mind wandering, are related, but also emphasized substantial heterogeneity across studies (Cárdenas-Egúsquiza & Berntsen, 2022). Our findings add to this literature by suggesting that, in a clinical adult ADHD sample, the timing and subjective quality of sleep may be more relevant to excessive mind wandering than the amount of sleep reported. This does not imply that sleep duration is unimportant; rather, it indicates that sleep duration alone may not adequately capture the sleep-related processes linked to internally generated cognitive activity in ADHD.

A possible interpretation is that delayed sleep timing and poor subjective sleep quality may increase vulnerability to excessive mind wandering by weakening cognitive control and arousal regulation. Adult ADHD has been associated with altered coordination between executive control networks and the default mode network, including insufficient suppression of internally oriented processes during externally directed tasks (Bozhilova et al., 2018; Cortese et al., 2012; Rubia, 2018). Circadian misalignment and suboptimal sleep quality may further compromise prefrontal control and increase the likelihood of task-unrelated internal mentation. This interpretation is compatible with existing neurocognitive models, but remains speculative in the present study because no objective sleep, circadian, or neuroimaging measures were obtained.

The association between mid-sleep time and excessive mind wandering was observed after accounting for anxiety–depressive symptom burden. This is important because affective symptoms are common in adult ADHD and are themselves associated with both sleep disturbance and repetitive or intrusive cognition (Fu et al., 2025; Katzman et al., 2017). Nevertheless, adjustment for HADS total score does not fully account for psychiatric comorbidity. The ADHD group was clinically heterogeneous, and many participants had comorbid psychiatric conditions or were receiving ADHD medication. This increases the ecological validity of the sample, but also introduces potential residual confounding. Medication status, dose, treatment duration, adherence, and timing of medication intake were not modelled in detail and may have influenced sleep–wake patterns or cognitive symptoms, as suggested in previous studies (Hvolby, 2015).

From a clinical perspective, these findings suggest that assessment of sleep in adult ADHD should not be limited to sleep duration. Sleep timing, delayed sleep phase tendencies, irregular sleep–wake schedules, and subjective sleep quality may be particularly relevant when evaluating patients who report excessive mind wandering, cognitive overactivity, or fluctuating attention across the day (Bijlenga et al., 2019; Wajszilber et al., 2018). Routine clinical assessment may therefore benefit from brief but systematic questions about bedtime, wake time, mid-sleep timing, weekday–weekend shifts, morning difficulty, and evening alertness. The present findings do not establish that modifying sleep timing will reduce excessive mind wandering, but they provide a rationale for future studies testing whether interventions targeting circadian alignment and sleep quality can improve internally driven attentional dysregulation in adult ADHD (Surman & Walsh, 2021; Walter et al., 2026).

Several strengths and limitations should be noted. Strengths include the relatively large clinical ADHD sample, the inclusion of a screened control group, structured psychiatric assessment, and the use of validated self-report measures. The study also addressed a clinically relevant but underexamined question: whether sleep timing is associated with excessive mind wandering beyond sleep duration and subjective sleep quality. However, the cross-sectional design precludes causal inference. Subjective sleep quality was assessed using a study-specific single-item measure rather than a validated multidimensional sleep questionnaire; this may have limited measurement precision and represents a clear limitation of the study. Future studies should employ validated sleep instruments to provide a more comprehensive and reliable assessment. Sleep variables were based on self-report, and mid-sleep time was derived from reported sleep onset and awakening times rather than from actigraphy, sleep diaries, or biological circadian markers such as dim-light melatonin onset. Social jetlag could not be assessed because weekday and weekend sleep schedules were not evaluated separately, limiting the interpretation of variability in sleep timing.

The study was also not designed to examine presentation-specific or sex-stratified differences in excessive mind wandering or its associations with sleep-related characteristics. Because the primary aim was to evaluate the independent association of diagnostic status with excessive mind wandering after adjustment for demographic, affective, and sleep-related variables, the regression analysis was conducted in the pooled sample. Although this modelling strategy was consistent with the primary research question, it did not allow potentially distinct multivariable patterns within the ADHD and control groups to be evaluated separately. Finally, psychiatric comorbidities and medication-related variables could not be fully accounted for, and objective measures of attention or mind wandering could not be used.

## 5. Conclusions

Excessive mind wandering in adult ADHD was associated with ADHD symptom severity, anxiety–depressive symptom burden, subjective sleep quality, and sleep timing. Later mid-sleep time, but not total sleep duration, remained independently associated with excessive mind wandering. These findings suggest that the timing and quality of sleep may be clinically relevant to internally generated attentional dysregulation in adult ADHD. Future longitudinal and experimental studies using objective sleep and circadian measures are needed to clarify whether delayed sleep timing contributes to excessive mind wandering and whether targeting sleep–wake regulation can reduce cognitive symptoms in adults with ADHD.

## Figures and Tables

**Table 1:** Demographic, clinical, sleep-related, and self-report characteristics of adults with ADHD and healthy controls.

Variable	ADHD (n = 316)	Control (n = 121)	t/χ^2^	*p*	Cohen’s d
Age (years)	24.23 ± 6.50	25.26 ± 7.52	−1.48	0.140	0.16
Sex, n (%)			χ^2^ = 2.63	0.105	—
Female	166 (52.5%)	74 (61.2%)			
Male	150 (47.5%)	47 (38.8%)			
Duration of education (years)	14.68 ± 2.73	15.83 ± 2.51	−3.83	<0.001	0.43
ASRS	50.45 ± 9.21	25.92 ± 11.41	20.74	<0.001	2.30
MEWS	26.02 ± 5.68	13.64 ± 6.70	18.89	<0.001	1.95
HADS total	24.67 ± 9.40	15.81 ± 8.35	9.38	<0.001	0.90
Subjective sleep quality	4.07 ± 2.37	5.54 ± 2.36	−5.83	<0.001	0.56
Average sleep duration (h)	6.72 ± 1.65	6.60 ± 1.27	0.19	0.850	0.02
Mid-sleep time (h)	5.41 ± 2.11	4.28 ± 1.20	6.41	<0.001	0.60

Note: Values are presented as mean ± standard deviation or n (%), as appropriate. Independent samples *t*-tests were used for continuous variables, and chi-square tests were used for categorical variables. Effect sizes were reported using Cohen’s d and interpreted as small (0.20–0.49), medium (0.50–0.79), and large (≥0.80). ADHD = Attention-Deficit/Hyperactivity Disorder; ASRS = Adult ADHD Self-Report Scale; MEWS = Mind Excessively Wandering Scale; HADS = Hospital Anxiety and Depression Scale.

**Table 2:** Pearson correlations among demographic, clinical, sleep-related, and self-report variables in the ADHD and control groups.

Variable	1	2	3	4	5	6	7	8
1. Age	—	0.55 **	0.03	0.04	0.01	−0.02	0.03	−0.07
2. Duration of education	0.07	—	0.07	0.05	0.12 *	−0.02	−0.04	−0.03
3. ASRS	−0.23 *	0.08	—	0.30 **	0.57 **	−0.27 **	−0.06	0.16 **
4. HADS total	−0.17	0.19 *	0.38 **	—	0.26 **	−0.32 **	−0.13 *	0.15 **
5. MEWS	−0.30 **	0.05	0.79 **	0.47 **	—	−0.18 **	0.00	0.15 **
6. Subjective sleep quality	0.21 *	0.12	−0.36 **	−0.34 **	−0.41 **	—	0.21 **	−0.36 **
7. Average sleep duration	0.14	−0.06	−0.04	−0.11	−0.09	0.37 **	—	0.06
8. Mid-sleep time	−0.27 **	−0.12	0.28 **	0.22 *	0.38 **	−0.45 **	−0.05	—

Note: The upper triangle presents Pearson correlation coefficients for the ADHD group, and the lower triangle presents those for the control group * *p* < 0.05; ** *p* < 0.01 (two-tailed). ADHD: Attention-Deficit/Hyperactivity Disorder; ASRS: Adult ADHD Self-Report Scale; MEWS: Mind Excessively Wandering Scale; HADS: Hospital Anxiety and Depression Scale.

**Table 3:** Between-group comparisons of correlations between MEWS and clinical and sleep-related variables.

Correlation	ADHD, r	Control, r	Fisher’s z	*p*
MEWS–ASRS total	0.57	0.79	−3.92	<0.001
MEWS–HADS total	0.26	0.47	−2.26	0.024
MEWS–subjective sleep quality	−0.18	−0.41	2.35	0.019
MEWS–mid-sleep time	0.15	0.38	−2.30	0.021

Note: Between-group differences in independent correlation coefficients were tested using Fisher’s r-to-z transformation. Negative z values indicate stronger positive correlations in the control group, whereas the positive z value for subjective sleep quality indicates a stronger negative correlation in the control group. ASRS = Adult ADHD Self-Report Scale; HADS = Hospital Anxiety and Depression Scale; MEWS = Mind Excessively Wandering Scale.

**Table 4:** Multiple linear regression analysis predicting excessive mind wandering in the total sample.

Predictor	B	SE	β	t	*p*	95% CI for B	VIF
Group (ADHD = 1)	9.83	0.67	0.54	14.57	<0.001	8.50–11.15	1.27
Sex (male = 1)	−0.53	0.54	−0.03	−0.98	0.330	−1.60–0.54	1.02
Duration of education	0.09	0.09	0.03	0.97	0.335	−0.09–0.27	1.03
HADS total	0.17	0.03	0.21	5.34	<0.001	0.11–0.23	1.37
Subjective sleep quality	−0.36	0.13	−0.11	−2.71	0.007	−0.62–−0.10	1.47
Average sleep duration	0.16	0.18	0.03	0.88	0.381	−0.20–0.52	1.11
Mid-sleep time	0.30	0.15	0.07	1.99	0.048	0.003–0.60	1.27

Note: B = unstandardized coefficient; SE = standard error; β = standardized coefficient; CI = confidence interval; VIF = variance inflation factor. ADHD was coded as 1 and controls as 0; male sex was coded as 1 and female as 0. The model was statistically significant (F(7, 429) = 68.55, *p* < 0.001) and explained 52.0% of the variance in MEWS scores (adjusted R^2^ = 0.520). Statistical significance was set at *p* < 0.05. HADS: Hospital anxiety and depression scale.

## Data Availability

The datasets presented in this article are not readily available due to ethical and privacy restrictions, as the dataset contains participant-level clinical and questionnaire data and the informed consent did not include permission for unrestricted public data sharing. Requests to access the datasets should be directed to the corresponding author, Samet Kaya, at samet.kaya4@saglik.gov.tr or abdussamedkaya1994@gmail.com, and will be considered subject to approval by the relevant ethics committee and applicable data protection regulations.
